# Promoting physical activity with self-management support for those with multimorbidity: a randomised controlled trial

**DOI:** 10.3399/BJGP.2021.0172

**Published:** 2021-11-02

**Authors:** Kamlesh Khunti, Patrick J Highton, Ghazala Waheed, Helen Dallosso, Emma Redman, Mark E Batt, Melanie J Davies, Laura J Gray, Louisa Y Herring, Hamidreza Mani, Alex Rowlands, Tom Yates

**Affiliations:** Diabetes Research Centre, University of Leicester, Leicester.; Diabetes Research Centre, University of Leicester, Leicester.; Diabetes Research Centre, University of Leicester, Leicester.; NIHR Applied Research Collaboration East Midlands, Leicester.; Leicester Diabetes Centre, Leicester.; Arthritis Research UK Centre for Sport, Exercise and Osteoarthritis, Queens Medical Centre, Nottingham.; Diabetes Research Centre, University of Leicester, Leicester.; Department of Health Sciences, University of Leicester, Leicester.; NIHR Applied Research Collaboration East Midlands, Leicester.; Department of Diabetes and Endocrinology, Kettering General Hospital NHS Trust, Kettering.; Diabetes Research Centre, University of Leicester, Leicester.; Diabetes Research Centre, University of Leicester, Leicester.

**Keywords:** chronic disease, disease self-management, exercise, multimorbidity, physical activity, primary care

## Abstract

**Background:**

Targeted self-management programmes may improve health and increase physical activity (PA) in people with multimorbidity.

**Aim:**

To investigate the impact of a structured, theoretically driven, self-management group education programme on habitual PA levels in people with multimorbidity.

**Design and setting:**

Individually randomised controlled trial with 12-month follow-up, involving nine primary care practices in Leicestershire, UK.

**Method:**

In total, 353 adults with multimorbidity (age 67.8 years [±9 years], 161 male sex) were randomised to intervention (*n* = 180) or control (*n* = 173) groups. Intervention participants were invited to attend four group-based self-management sessions, centred primarily on increasing PA, and received motivational text-message support. The primary outcome measure was change in overall volume (time and intensity) of daily PA at 12 months, as measured by the GENEActiv wrist-worn accelerometer device.

**Results:**

At baseline, the total sample achieved 22 min of moderate–vigorous intensity PA per day (mean/participant). At 12 months, in the complete-case analysis, a reduction in daily mean PA volume was seen in the intervention group relative to control (−0.80 milligravity [m *g*]; 95% confidence interval [CI] = −1.57 to −0.03; *P* = 0.04). Reductions were also seen in the intervention group in time spent in moderate–vigorous PA (−3.86 min per day; 95% CI= −6.70 to −1.03; *P* = 0.008) and time spent at an intensity equivalent to a slow walk (−4.66 min per day; 95% CI = −8.82 to −0.51; *P* = 0.028). However, the per-protocol analysis (excluding participants who did not attend at least one education session) found no between-group differences in overall daily PA at 12 months (−0.65 mg; 95% CI = −1.46 to 0.15; *P* = 0.11).

**Conclusion:**

The self-management programme elicited a slight reduction in PA levels in people with multimorbidity. Future research should identify and target subgroups of those with multimorbidity in greatest need of PA promotion in order to maximise potential capacity for benefit, and also focus on refining the intervention in order to increase efficacy in increasing PA.

## INTRODUCTION

Multimorbidity, defined as the coexistence of ≥2 chronic conditions,^1^^,^^2^ is a growing global health concern that is highly prevalent in primary care and older populations.^3^^,^^4^ In the UK in 2015, 54% of those aged >65 years had multimorbidity; this is expected to reach 68% by 2035.^4^ Multimorbidity increases mortality, with an additive effect for each additional condition;^5^ it is also associated with reduced quality of life,^6^ physical function,^7^ and risk of depression.^8^ Populations with multimorbidity have high hospitalisation rates, healthcare usage, and healthcare costs, which increase with each additive condition.^9^

Disease self-management — whereby an individual takes responsibility for all, or some, aspects of their day-to-day disease management^10^ — promotes engagement and empowerment, and is recommended by the World Health Organization.^11^ Previous theory-driven, self-management interventions incorporating a variety of methodologies (for example, small group sessions, eHealth and/or mHealth support, printed materials) have successfully improved quality of life and self-efficacy in people with a variety of chronic diseases (such as diabetes, cardiovascular disease [CVD], arthritis),^12^ and other clinical outcomes in type 2 diabetes (T2DM).^13^^,^^14^ However, interventions designed specifically for multimorbidity are under-investigated and, therefore, are a research priority,^15^ holding significant promise for improving health outcomes.

People with multimorbidity typically display low physical activity (PA) levels,^7^ which are associated with worsened all-cause mortality,^16^ disease burden,^16^ health-related quality of life,^17^ physical function,^18^ and mental health.^19^ Consequentially, PA levels display an inverse dose–response relationship with multimorbidity burden^20^ and mortality.^21^ In people with CVD and pre-diabetes, increasing PA by 2000 steps per day is associated with an 8% reduction in the risk of cardiovascular events, irrespective of changes in body mass;^22^ however, to the authors’ knowledge, no studies have developed or tested an intervention designed specifically to increase PA levels in people with multimorbidity. The aim of this study was to evaluate a programme in a primary care setting that targets PA, other lifestyle factors, and disease self-management in people with multimorbidity.

## METHOD

### Study design and recruitment

A detailed study protocol (ISRCTN42791781) has been published elsewhere.^23^ The study was a single-site, two-arm, parallel-group, open-label, 12-month randomised controlled trial (RCT) testing the effectiveness of a structured, self-management programme in participants with multimorbidity who were recruited from primary care practices in Leicestershire, UK. Potential participants were identified from a search of each practice’s healthcare records. Following confirmation of eligibility from a practice staff member, eligible patients were sent a study invitation pack with a reply slip, after which a study team member contacted the patients to arrange a baseline visit and take informed consent. Ethical approval was obtained and participants provided written informed consent before data were collected.

**Table:** How this fits in

People with multimorbidity typically display increased morbidity and mortality risk, driven in part by reduced levels of habitual physical activity (PA). Disease self-management empowers patients to take more of an active role in their own health care and has shown promise in individual conditions, but is under-researched in multimorbidity. This study investigated the impact of a targeted, group-based, disease self-management programme on habitual PA levels in people with multimorbidity. A slight decrease in PA levels was observed, suggesting that the intervention was ineffective, and that future research should focus on how to identify those at greatest need for PA intervention, and how to refine the delivered intervention in order to increase efficacy in increasing PA in those with multimorbidity.

### Inclusion and exclusion criteria

Multimorbidity was defined as the coexistence of ≥2 chronic conditions^1^ listed in the Quality and Outcomes Framework clinical domains^24^ (Supplementary Box S1). Other inclusion criteria were:

- aged 40–85 years;
- a good understanding of English;
- able to give written informed consent;
- access to a mobile phone; and
- able to walk independently.

Exclusion criteria were:

- pregnancy;
- current or recent (defined as <12 weeks) participation in another interventional trial; or
- frailty, according to certain criteria (outlined in Supplementary Box S2) — this was for safety reasons relating to the promotion of unsupervised PA.

### Randomisation and blinding

Participants were individually randomised (1:1), stratified by sex and ethnicity (white European; other), as per a computer-generated randomisation sequence using a variable block size by an independent researcher. Research nurses were unaware of allocation during data-collection visits; analysis of the accelerometer device that was used to track PA was completed by a blinded researcher. Participants and the central study team could not be blinded.

### Treatment

Participants in the control group continued to receive their usual disease management as required, which was unaffected by their participation in the trial. Intervention participants not only continued to receive their usual disease management as required from their clinician(s), but also received the intervention as explained below.

### Intervention

The Movement through Active Personalised engagement (MAP) programme^23^ was iteratively developed, tested, and refined using literature reviews, patient and public coproduction, and focus groups with people with multimorbidity, their relatives, and primary care clinicians and nurses. The focus groups informed the programme content to include both a focus on PA and to cover generic self-management challenges. The intervention was underpinned by social learning theory.^25^ A draft programme was iteratively tested and modified with patient volunteers prior to finalisation.

The final programme comprised four, 1.5-hour group sessions with person-centred^26^ self-monitoring and goal setting, delivered by a trained facilitator at 2-week intervals in local community settings. Sessions were delivered concurrently, and participants were able to attend different venues if convenient. The sessions not only focused primarily on increasing PA, but also addressed key non-disease-specific self-management challenges and themes (mastering emotions, managing treatments, communication within health care). Programme delivery and fidelity were assessed using a structured observation tool.^27^^,^^28^ To support a long-term change in health behaviour, regular reminder and motivational text messages were sent to the participants in the intervention arm; the frequency and content of these were fixed for all members of the intervention group and based on previous successful self-management programmes.^29^^–^31 Frequency was daily during the 8 weeks when the group sessions were delivered and for 2 weeks after the 6-month timepoint had been reached; this reduced to three times per week over the remainder of the 12-month period (Supplementary Figure S1).

Increasing PA was further facilitated and encouraged by providing resistance bands in order to complete resistance training at home and pedometers to help track PA. Demonstration of how to use this equipment was provided.

### Primary outcome

The primary outcome was a change in the volume of daily PA from baseline to 12 months, measured using the GENEActiv wrist-worn, tri-axial accelerometer (Activinsights Ltd, Cambridgeshire, UK); this measured average movement intensity in milligravitational units (m *g*), which was indicative of the overall volume of daily PA, using the Euclidean norm minus *g* method.^32^ Participants wore the accelerometer for 8 consecutive days at baseline, 6 months, and 12 months. Data were included if participants recorded ≥1 valid days (that is, ≥16 hours per day) and were analysed using an open-source *R*-package, GGIR).^33^ To translate findings from milligravitational units, the number of minutes of brisk walking that would lead to observed changes in average daily acceleration — based on a change of 0.8 m *g* representing a change of 5 min’ brisk walking — were determined.^34^

### Secondary outcomes

PA-related secondary outcomes were determined from the accelerometer data. Other secondary outcome measures were taken at baseline and 12 months; these included clinical, venous blood (lipid profile, kidney function, glycated haemoglobin [HbA1c]), and anthropometric measures.

The following questionnaires were completed at baseline and at 12 months:

- Recent Physical Activity Questionnaire;^35^
- Adherence Starts with Knowledge 12;^36^
- EuroQol five-dimensional, five-level version (EQ-5D-5L);^37^
- Hospital Anxiety and Depression Scale;^38^
- Chronic Disease Self-Efficacy Scale;^39^ and
- Self-Efficacy for Exercise scale.^40^

Dietary behaviour was conducted based on questionnaires developed previously.^41^ Participants in the intervention group also completed a questionnaire following each session to assess the perception of that session and its potential for benefit.

### Sample size

In order to detect a minimum clinically important difference (MCID) in mean volume of daily PA of 2.1 m *g* at 12 months *,* and assuming a standard deviation (SD) of 5.3 m *g,*^42^ power of 80%, and a statistical significance level of 5%, a total of 202 participants were required. Allowing for 20% loss to follow-up and 20% non-compliance of accelerometer/intervention attendance meant that at least 338 participants were required (169 per group).^23^ The value of 2.1 m *g* was chosen as it represents an increase in PA that is equivalent to walking at the threshold between light intensity and moderate intensity (for example, 4 km per hour) for 30 min per day or 10–15 min of brisk walking per day.^34^^,^^43^

### Statistical analysis

Binary and categorical baseline variables were presented by group as numbers (with percentages), and continuous variables as means (with SDs) or medians (with lower/upper quartiles).

The primary outcome was analysed using a linear regression model, with change in overall daily PA as the dependent variable and the randomisation group as an explanatory variable, adjusted for stratification factors (sex and ethnicity), change in wear time between baseline and 12 months, and baseline outcome value. The effect of ≥3 valid days of accelerometer wear time on the primary outcome was checked using a sensitivity analysis and similar methodology.

The main analysis was conducted using a modified intention-to-treat approach and participants with follow-up data were analysed in the group to which they were assigned. A sensitivity analysis was used to assess the impact of missing followup data using multiple imputation and to undertake a per-protocol analysis; only those participants in the intervention group who attended ≥1 education session were included. Multiple imputation was used to replace missing values with multiple sets of simulated values to allow for standard analysis on each completed dataset,^44^ followed by Rubin’s formula^45^ to combine the parameter estimates and standard errors into a single set of results. The imputation model included the variables that were included in the linear regression analysis.

Further exploratory analysis was completed on those who attended 1–3 sessions, versus four sessions, to assess intervention impact. Subgroup analyses were performed to examine the effects between the intervention arm and the following prespecified subgroups of baseline characteristics:

- age (<65 years and ≥65 years);
- sex;
- median number of comorbidities (<4 and ≥4);
- depression;
- arthritic condition;
- cardiovascular condition;
- T2DM; and
- PA (low and high).

The interaction effects between the treatment and subgroups were used to assess differences in outcome by subgroup. Secondary outcomes were analysed in a similar manner to the primary outcome. Statistical significance was assessed at the 5% level with 95% confidence intervals (CIs). All *P*-values were two sided and *P*<0.05 was considered statistically significant. Analyses were completed using Stata (version15).

## RESULTS

### Participants

**Figure 1.**
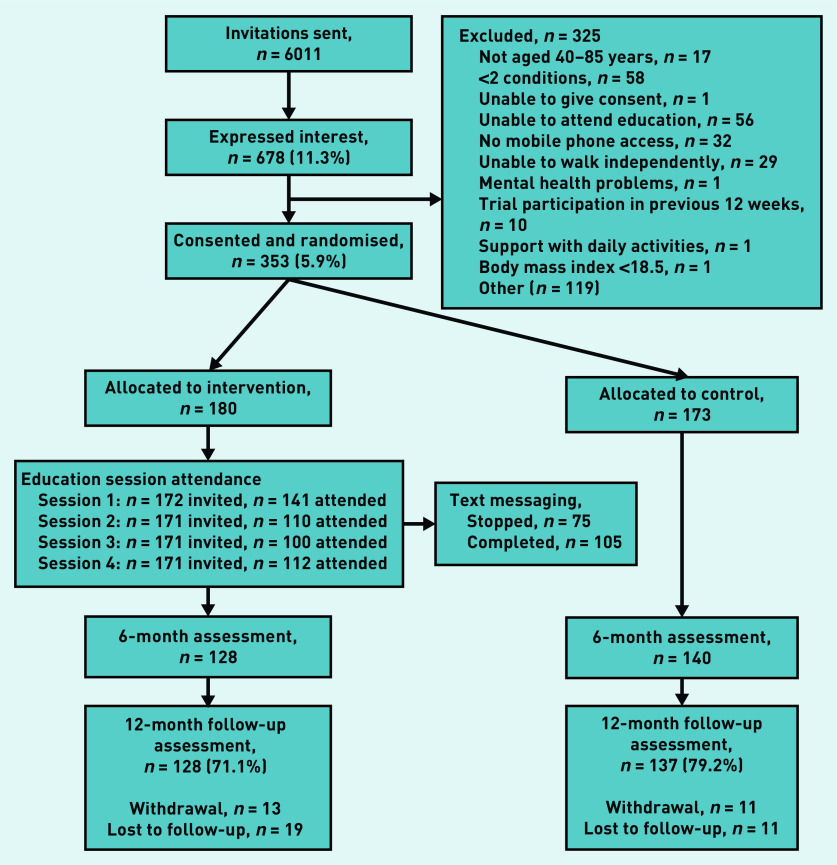
*Flowchart of sample recruitment, allocation, and study completion.*

In total, 6011 people from nine general practices were invited to participate in the study. Out of 678 (11.3%) expressions of interest, 353 (5.9% of all invited) individuals were eligible, consented to take part, and were randomised to either the intervention (*n* = 180) or control (*n* = 173) group (Figure 1).

Participant baseline characteristics are presented in Supplementary Table S1. The mean number of conditions was 4.32. In total, 83.9% had a cardiovascular condition, 29.8% had a respiratory condition, 45.3% were obese, 57.5% had a condition classified as ‘high dependency’ (which includes diabetes mellitus), 35.4% had depression, and 22.7% had a musculoskeletal condition.

### Intervention attendance and receipt

Of the 180 participants in the intervention group, 141 78.3% attended Session 1; attendance at Sessions 2–4 was 110 (61.1%), 100 (55.6%), and 112 (62.2%) respectively (Supplementary Table S2). In total, 75 people (42%) sent a request during the study period to stop receiving the text messages. Following the ‘moving more’ session (Session 1), 127 (90%) of participants agreed that moving more would benefit them, but only 93 (66%) reported that this opinion had changed in response to the intervention. Although 127 (86%) reported that they were considering a change in their PA habits, only 93 (66%) reported feeling able to do so.

### Physical activity

Primary outcome data were available for 128 (71.1%) and 137 (79.2%) of the intervention and control groups respectively. The participants undertook a mean moderate–vigorous PA of 22 min per day at baseline across both groups (Table S1). Although a reduction in overall daily PA was seen in both groups (Table 1), this was greater in the intervention group compared with the control group at 12 months, both in the main analysis (−0.80 m *g*; 95% CI = −1.57 to −0.03; *P* = 0.04) and in the intention-to-treat analysis (−0.83 m *g*; 95% CI = −1.61 to −0.04; *P* = 0.03). However, the per-protocol analysis (which excluded participants who did not attend at least one education session) found no between-group differences in overall daily PA at 12 months (−0.65 m *g*; 95% CI = −1.46 to 0.15; *P* = 0.11) (Table 1). There was no effect of adjusting for same-household clustering. The relative reduction of 0.8 m *g* in the intervention group approximates replacing 5 min’ brisk walking with sedentary time.^34^

**Table 1.** Changes in overall daily physical activity for at least 1 valid day of accelerometer wear at 12 months

**Analysis**	**Participants, *n***		**Mean change from baseline, m *g***		**Adjusted difference at follow-up^a^**	
		
	**Control, *n*= 173**	**Intervention, *n* = 180**	**Control**	**Intervention**	**Coefficient (95% CI)**	***P*-value**
**Modified intention to treat^b^**						
Overall daily physical activity	137	128	−0.36	−1.20	−0.80 (−1.57 to −0.03)	0.04
Adjusted for same-household clustering					−0.80 (−1.59 to −0.02)	0.05^c^

**Intention to treat^d^**						
Overall daily physical activity	173	180	−0.35	−1.13	−0.83 (−1.61 to −0.04)	0.03
Adjusted for same-household clustering					−0.83 (−1.61 to −0.04)	0.04

**Per protocol^e^**						
Overall daily physical activity	137	111	−0.36	−1.06	−0.65 (−1.46 to 0.15)	0.11
Adjusted for same-household clustering					−0.65 (−1.46 to 0.16)	0.11

a *Adjusted for stratification factors: sex, ethnicity; change from baseline in accelerometer wear time, and baseline value of outcome.*

b *Participants with missing outcome data or missing variables required for the model adjustment were excluded.*

c *= 0.045.*

d *Missing data were imputed using multiple imputation.*

e *Participants who did not engage with at least one group session of the programme have been excluded from the intervention arm. CI = confidence interval.*

Sensitivity analyses based on the minimum-wear criteria of 3 days showed comparable results (−0.85 m *g*; 95% CI = −1.63 to 0.08; *P* = 0.03) (Supplementary Table S3). No statistically significant effects were observed in the subgroup analyses (Supplementary Figure S2).

Analysis of PA-related secondary outcomes showed a relative reduction in time spent undertaking moderate–vigorous PA (−3.86 min per day; 95% CI = −6.70 to −1.03; *P* = 0.008) and time spent at an intensity equivalent to a slow walk (−4.66 min per day; 95% CI = −8.82 to −0.51; *P* = 0.028) in the intervention group at 12 months (Table 2). No other statistically significant findings were observed (Table 2), and no effects were observed in the exploratory analysis of session attendance (Supplementary Table S4).

**Table 2.** Changes in secondary outcomes related to physical activity for at least 1 valid day of accelerometer wear at follow-up

**Physical activity-related secondary outcome**	**Participants, *n***		**Mean change from baseline**		**Adjusted difference at follow-up^a^**	
		
	**Control, *n*= 173**	**Intervention, *n*= 180**	**Control**	**Intervention**	**Coefficient (95% CI)**	***P*-value**
**Overall daily physical activity, m*g***						
6 months	140	128	−0.38	−0.36	0.12 (−0.80 to 1.04)	0.793

**Average acceleration for most active continuous 30 min, m*g***						
6 months	140	128	−1.17	−1.68	1.07 (−3.07 to 5.21)	0.611
12 months	137	128	1.11	−4.17	−3.71 (−7.97 to 0.54)	0.087

**Minutes per day spent in moderate-to-vigorous 1-min bouts of physical activity (min/day)**						
6 months	140	128	−1.76	0.01	1.88 (−1.53 to 5.28)	0.279
12 months	137	128	0.54	−3.72	−3.86 (−6.70 to −1.03)	0.008

**Minutes per day spent in slow walk, classified as**>**100 m*g* (min/day)**						
6 months	140	128	−2.29	−1.04	1.70 (−3.46 to 6.86)	0.518
12 months	137	128	−1.09	−6.23	−4.66 (−8.82 to −0.51)	0.028

**Minutes per day spent in brisk walk, classified as**>**200 m*g* (min/day)**						
6 months	140	128	0.06	0.001	0.23 (−1.44 to 1.90)	0.789
12 months	137	128	0.10	−1.40	−1.23 (−2.81 to 0.35)	0.127

**Minutes per day spent sedentary/inactive (min)**						
6 months	135	121	1.13	2.71	−1.78 (−19.33 to 15.77)	0.842
12 months	136	124	−10.15	5.22	9.91 (−11.78 to 31.60)	0.369

**Minutes’ sleep duration per night (min)**						
6 months	135	121	−2.20	−2.80	0.69 (−10.49 to 11.86)	0.904
12 months	136	124	5.63	2.43	−1.33 (−16.98 to 14.32)	0.867

a *Adjusted for stratification factors: sex, ethnicity; change from baseline in accelerometer wear time and baseline value of outcome. Participants with missing outcome data or missing variables required for the model adjustment were excluded. CI = confidence interval.*

### Anthropometric and clinical measures

No statistically significant differences between groups were observed in anthropometric or clinical measures (Table 3).

**Table 3.** Changes in anthropometric and clinical measures at 12 months

**Anthropometric/clinical measure**	**Participants, *n***		**Mean change from baseline**		**Adjusted difference at follow-up^a^**	
		
	**Control, *n*= 173**	**Intervention, *n*= 180**	**Control**	**Intervention**	**Coefficient (95% CI)**	***P*-value**
Body mass index, kg/m^2^	130	123	−0.19	0.002	0.18 (−0.10 to 0.47)	0.205
Body weight, kg	130	123	−0.85	−0.33	0.51 (−0.26 to 1.28)	0.192
Waist circumference, cm	130	123	0.61	0.34	−0.33 (−1.84 to 1.19)	0.671
Hip circumference, cm	130	123	−0.12	0.19	0.08 (−1.29 to 1.44)	0.914
Waist:hip ratio	130	123	0.01	0.001	0.005 (−0.01 to 0.02)	0.629
Left-hand grip, kg	130	123	−2.97	−1.57	1.06 (−0.51 to 2.64)	0.186
Right-hand grip, kg	129	123	−2.60	−1.59	0.78 (−0.87 to 2.43)	0.352
Systolic blood pressure, mm HG	130	123	−2.71	−1.21	1.61 (−2.03 to 5.25)	0.385
Diastolic blood pressure, mm HG	130	123	−3.61	−3.81	−0.05 (−2.03 to 1.94)	0.962
Resting heart rate, b.p.m.	130	123	0.12	−0.59	−0.65 (−2.57 to 1.26)	0.503
Total cholesterol, mmol/l	124	125	−0.18	−0.24	−0.04 (−0.19 to 0.11)	0.585
HDL cholesterol, mmol/l	124	125	−0.02	−0.01	−0.02 (−0.10 to 0.05)	0.540
LDL cholesterol, mmol/l	123	122	−0.13	−0.23	−0.07 (−0.20 to 0.05)	0.226
Triglycerides, mmol/l	124	125	−0.15	0.003	0.16 (−0.01 to 0.32)	0.058
Total cholesterol:HDL ratio	124	125	−0.13	−0.19	−0.01 (−0.11 to 0.10)	0.925
HbA1c, %	129	129	−0.09	−0.08	−0.001 (−0.11 to 0.11)	0.985
HbA1c, mmol/mol	129	129	−0.96	−1.24	−0.36 (−1.66 to 0.95)	0.591

a *Adjusted for stratification factors: sex, ethnicity; baseline value. Participants with missing outcome data or missing variables required for the model adjustment were excluded. CI = confidence interval. HbA1c = glycated haemoglobin. HDL = high-density lipoprotein. LDL = low-density lipoprotein.*

### Questionnaires

Questionnaire results are presented in Supplementary Table S5. The dietary questionnaire showed an increase relative to control in the consumption of fresh fruit in the intervention group at 12 months (0.30; 95% CI = 0.06 to 0.54; *P* = 0.02) — this was the only identifiable improvement. There was a statistically significant reduction relative to control at 12 months in self-rated health (visual analogue scale [VAS]) from the EQ-5D-5L (−3.85; 95% CI = −7.60 to −0.09; *P* = 0.045) in the intervention group. No differences were observed in other measures of quality of life, depression, anxiety, or disease, or exercise self-efficacy.

### Adverse events

In total, 100 adverse events were reported in the control (*n* = 48) and intervention (*n* = 52) groups during the study. Six of these (two in the control and four in the intervention groups) involved a non-elective overnight stay in hospital and were, therefore, described as serious adverse events. These were reported to the sponsor as part of the safety monitoring procedure and one resulted in the participant (who had terminal cancer) discontinuing their participation in the study. Details of the adverse events are provided in Supplementary Tables S6A and S6B.

## DISCUSSION

### Summary

In response to a structured, theoretically driven, self-management group education programme designed to increase habitual PA levels, a reduction was observed in overall volume of daily PA and self-reported health in both groups, but this was greater in the intervention group. Other than an increase in self-reported intake of fresh fruit in the intervention group, no other statistically significant effects were observed.

### Strengths and limitations

This is the first RCT, of which the authors are aware, to investigate the impact of self-management on device-measured, habitual PA in people with multimorbidity. Furthermore, the broad inclusion criteria increased external validity by increasing applicability to the heterogeneous multimorbid population, as the results may be applied to a large proportion of the population. However, although the study recruited to target and the 12-month follow-up rate was high, overall participation was lower than in a previous trial in this population,^46^ and may have introduced some recruitment bias, though as these studies investigated different interventions it is difficult to compare uptake rates. Furthermore, participants were not screened for PA level prior to randomisation. Given that participation bias is common in PA research,^47^ this may have introduced some PA-related selection bias.^47^ Lastly, the delay between the final education session and the next data collection timepoint (6 months) may have meant that any improvements in PA were missed because of a return to baseline levels.

### Comparison with existing literature

Although an age-related decline in PA levels over a 12-month period may be expected in this age group,^48^ the intervention group displayed a 0.8 m *g* reduction relative to control that equated, approximately, to replacing 5 min of brisk walking with sedentary time.^49^ Since the study conception, evidence suggests that an increase of only 500 steps per day — equivalent to approximately 5 min’ brisk walking — is associated with a decrease in CVD risk and mortality,^50^^–^52 with 0.8–1.0 m *g* being considered the MCID.^34^ As such, the reduction in PA levels observed in the intervention group, although statistically significant, was at the lower limit of what could be considered clinically meaningful. Furthermore, when analysing only those who attended the intervention, a smaller non-clinically significant reduction in PA was observed relative to control; it is, therefore, possible that the reduced levels of PA in the intervention group were a chance finding and that they were too low in magnitude to be considered potentially harmful.

The participants, on average, undertook 22 min of moderate–vigorous PA per day at baseline and were, therefore, meeting the government-recommended target of 150 min of moderate–vigorous PA per week,^53^ which may have limited the capacity for improvement. However, previous research revealed that people with multimorbidity spent less time engaged in moderate–vigorous PA (13 min per day)^54^ than observed here, but had a similar level of overall daily PA; this suggests that the participants of the study presented here may have completed more intentional PA than the general population with multimorbidity. As only 11.3% of those invited to participate in the study expressed an interest in doing so, and 52% of those were randomised, there may have been a disproportionate recruitment of participants who were already physically active.

Intervention attendance was facilitated by holding sessions in local community venues. However, the decreased attendance from 78% at Session 1 to 62% at Session 4 may, partly, explain the lack of intervention-induced improvement. However, this attendance rate is similar to a previous trial that investigated patient-centred care and disease self-management in multimorbidity;^46^ this highlights the difficulties in sustaining engagement in a population that often displays complex and diverse barriers to self-management.^55^

Nonetheless, none of these findings explain why a reduction in PA was observed in the intervention group relative to control. The findings of the per-protocol analysis suggest that non-attendees may have influenced the findings (although not being the primary analysis, this should be interpreted with caution). Intervention non-attendance may have been caused by factors that would lower PA levels, such as worsening of disease severity or mobility problems, which would also explain the reduction in VAS score; however, the VAS score reduction was smaller than the MCID of 7–10 reported for other chronic conditions.^56^^–^59 The negative result is surprising and does not follow the pattern of results seen in previous similar research in single-disease states;^12^^–^14 however, as mentioned, it is possible that this is a chance finding.

### Implications for research and practice

It is clear that this intervention was not successful at improving PA levels in this population. This highlights that broad self-management interventions may be ineffective at increasing PA in the diverse population with multimorbidity, who may well have diverse barriers, motivators, and needs concerning PA and health. It is possible that complexities surrounding variations or combinations of morbidity clustering contributed to this unexpected finding, though this trial was not designed or powered to investigate this. As physical activity promotion in multimorbidity is a relatively novel research area, there is limited previous research to which the authors can compare these findings, and as such further investigated is warranted. It is also possible that elements of the intervention may have limited its efficacy, for instance, focusing on a wide variety of disease self-management themes, while logical in this diverse population, might have diluted the message with regards to PA promotion. Similarly, other elements of the intervention, for example, the text messages, may have had limited or negative impact. Embedding a process evaluation within the intervention development, as recommended by the Medical Research Council framework for complex intervention development and evaluation,^60^ would also be useful to investigate this. Future research should also focus on identifying and targeting subgroups of people who are most in need of PA intervention, such as those with low baseline PA levels. This could be accomplished via a preliminary screening stage, where resources allow, and by involving those with low PA levels in the design of recruitment strategies in order to better target this population when recruiting for trials.

Conversely, future complex and theory-driven interventions could be developed with a realist evaluation approach^61^ or process evaluation, allowing for investigation into what worked for whom, as well as identifying potential causal mechanisms. Given the heterogeneity of the population with multimorbidity (that is, concerning comorbidity clustering and PA needs), this would allow for improved intervention personalisation. Health professionals could then use this information to promote regular PA and other self-management techniques in patients known to have the greatest need and capacity for benefit, as well as delivering more focused and tailored intervention.

In order to encourage attendance and sustained engagement in self-management programmes, it is important that there is direct communication between health professionals and patients;^62^ when practitioners have a detailed knowledge of the programme, they will be more likely to encourage their patients to attend as they will be better placed to support their patients to make informed decisions.^63^

## References

[1] World Health Organization (2016). Multimorbidity: technical series on safer primary care.

[2] Academy of Medical Sciences Advancing research to tackle multimorbidity: the UK and LMIC perspectives.

[3] Vetrano DL, Calderón-Larrañaga A, Marengoni A (2018). An international perspective on chronic multimorbidity: approaching the elephant in the room. J Gerontol A Biol Sci Med Sci.

[4] Kingston A, Robinson L, Booth H (2018). Projections of multi-morbidity in the older population in England to 2035: estimates from the Population Ageing and Care Simulation (PACSim) model. Age Ageing.

[5] Willadsen TG, Siersma V, Nicolaisdóttir DR (2018). Multimorbidity and mortality: a 15-year longitudinal registry-based nationwide Danish population study. J Comorb.

[6] Kanesarajah J, Waller M, Whitty JA, Mishra GD (2018). Multimorbidity and quality of life at mid-life: a systematic review of general population studies. Maturitas.

[7] Steeves JA, Shiroma EJ, Conger SA (2019). Physical activity patterns and multimorbidity burden of older adults with different levels of functional status: NHANES 2003–2006. Disabil Health J.

[8] Read JR, Sharpe L, Modini M, Dear BF (2017). Multimorbidity and depression: a systematic review and meta-analysis. J Affect Disord.

[9] Glynn LG, Valderas JM, Healy P (2011). The prevalence of multimorbidity in primary care and its effect on health care utilization and cost. Fam Pract.

[10] Lorig KR, Holman H (2003). Self-management education: history, definition, outcomes, and mechanisms. Ann Behav Med.

[11] Slama-Chaudhry A, Golay A (2019). Patient education and self-management support for chronic disease: methodology for implementing patient-tailored therapeutic programmes. Public Health Panorama.

[12] Allegrante JP, Wells MT, Peterson JC (2019). Interventions to support behavioral self-management of chronic diseases. Annu Rev Public Health.

[13] Crasto W, Jarvis J, Khunti K (2011). Multifactorial intervention in individuals with type 2 diabetes and microalbuminuria: the Microalbuminuria Education and Medication Optimisation (MEMO) study. Diabetes Res Clin Pract.

[14] Davies MJ, Heller S, Skinner TC (2008). Effectiveness of the diabetes education and self management for ongoing and newly diagnosed (DESMOND) programme for people with newly diagnosed type 2 diabetes: cluster randomised controlled trial. BMJ.

[15] Bratzke LC, Muehrer RJ, Kehl KA (2015). Self-management priority setting and decision-making in adults with multimorbidity: a narrative review of literature. Int J Nurs Stud.

[16] Lee I-M, Shiroma EJ, Lobelo F (2012). Effect of physical inactivity on major noncommunicable diseases worldwide: an analysis of burden of disease and life expectancy. Lancet.

[17] Bize R, Johnson JA, Plotnikoff RC (2007). Physical activity level and health-related quality of life in the general adult population: a systematic review. Prev Med.

[18] Yorston LC, Kolt GS, Rosenkranz RR (2012). Physical activity and physical function in older adults: the 45 and up study. J Am Geriatr Soc.

[19] Chekroud SR, Gueorguieva R, Zheutlin AB (2018). Association between physical exercise and mental health in 1.2 million individuals in the USA between 2011 and 2015: a cross-sectional study. Lancet Psychiatry.

[20] Dhalwani NN, O’Donovan G, Zaccardi F (2016). Long terms trends of multimorbidity and association with physical activity in older English population. Int J Behav Nutr Phys Act.

[21] Chudasama YV, Khunti KK, Zaccardi F (2019). Physical activity, multimorbidity, and life expectancy: a UK Biobank longitudinal study. BMC Med.

[22] Yates T, Haffner SM, Schulte PJ (2014). Association between change in daily ambulatory activity and cardiovascular events in people with impaired glucose tolerance (NAVIGATOR trial): a cohort analysis. Lancet.

[23] Dallosso H, Yates T, Mani H (2018). Movement through Active Personalised engagement (MAP) — a self-management programme designed to promote physical activity in people with multimorbidity: study protocol for a randomised controlled trial. Trials.

[24] Gillam SJ, Siriwardena AN, Steel N (2012). Pay-for-performance in the United Kingdom: impact of the quality and outcomes framework: a systematic review. Ann Fam Med.

[25] Bandura A (1977). Social learning theory.

[26] Coulter A, Entwistle VA, Eccles A (2015). Personalised care planning for adults with chronic or long-term health conditions.. Cochrane Database Syst Rev.

[27] Bryman A (2016). Social research methods.

[28] Skinner TC, Carey ME, Cradock S (2008). ‘Educator talk’ and patient change: some insights from the DESMOND (Diabetes Education and Self Management for Ongoing and Newly Diagnosed) randomized controlled trial. Diabet Med.

[29] Chen Z-W, Fang L-Z, Chen L-Y, Dai H-L (2008). Comparison of an SMS text messaging and phone reminder to improve attendance at a health promotion center: a randomized controlled trial. J Zhejiang Univ Sci B.

[30] de Jongh T, Gurol-Urganci I, Vodopivec-Jamsek V (2008). Mobile phone messaging telemedicine for facilitating self management of long-term illnesses.. Cochrane Database Syst Rev.

[31] Redfern J, Thiagalingam A, Jan S (2014). Development of a set of mobile phone text messages designed for prevention of recurrent cardiovascular events. Eur J Prev Cardiol.

[32] Van Hees VT, Gorzelniak L, Leon ECD (2013). Separating movement and gravity components in an acceleration signal and implications for the assessment of human daily physical activity. PLoS One.

[33] Migueles JH, Rowlands AV, Huber F (2019). GGIR: a research community–driven open source R package for generating physical activity and sleep outcomes from multi-day raw accelerometer data. J Meas Phys Behav.

[34] Rowlands A, Davies M, Dempsey P (2021). Wrist-worn accelerometers: recommending ∼1.0mg as the minimum clinically important difference (MCID) in daily average acceleration for inactive adults. Br J Sports Med.

[35] Besson H, Brage S, Jakes RW (2010). Estimating physical activity energy expenditure, sedentary time, and physical activity intensity by self-report in adults. Am J Clin Nutr.

[36] Matza LS, Park J, Coyne KS (2009). Derivation and validation of the ASK-12 adherence barrier survey. Ann Pharmacother.

[37] Herdman M, Gudex C, Lloyd A (2011). Development and preliminary testing of the new five-level version of EQ-5D (EQ-5D-5L). Qual Life Res.

[38] Zigmond AS, Snaith RP (1983). The Hospital Anxiety and Depression Scale. Acta Psychiatr Scand.

[39] Lorig K, Stewart A, Ritter P (1996). Outcome measures for health education and other health care interventions.

[40] Resnick B, Jenkins LS (2000). Testing the reliability and validity of the Self-Efficacy for Exercise scale. Nurs Res.

[41] Bingham SA, Gill C, Welch A (1997). Validation of dietary assessment methods in the UK arm of EPIC using weighed records, and 24-hour urinary nitrogen and potassium and serum vitamin C and carotenoids as biomarkers. Int J Epidemiol.

[42] Bell JA, Hamer M, van Hees VT (2015). Healthy obesity and objective physical activity. Am J Clin Nutr.

[43] Ainsworth BE, Haskell WL, Herrmann SD (2011). 2011 Compendium of Physical Activities: a second update of codes and MET values. Med Sci Sports Exerc.

[44] StataCorp Ltd (2021). Stata multiple-imputation reference manual.

[45] Rubin DB (1976). Inference and missing data. Biometrika.

[46] Salisbury C, Man M-S, Bower P (2018). Management of multimorbidity using a patient-centred care model: a pragmatic cluster-randomised trial of the 3D approach. Lancet.

[47] de Souto Barreto P, Ferrandez A-M, Saliba-Serre B (2013). Are older adults who volunteer to participate in an exercise study fitter and healthier than nonvolunteers? The participation bias of the study population. J Phys Act Health.

[48] Milanović Z, Pantelić S, Trajković N (2013). Age-related decrease in physical activity and functional fitness among elderly men and women. Clin Interv Aging.

[49] Rowlands AV, Edwardson CL, Davies MJ (2018). Beyond cut points: accelerometer metrics that capture the physical activity profile. Med Sci Sports Exerc.

[50] Yates T, Gray LJ, Henson J (2019). Impact of depression and anxiety on change to physical activity following a pragmatic diabetes prevention program within primary care: pooled analysis from two randomized controlled trials. Diabetes Care.

[51] Lee I-M, Shiroma EJ, Kamada M (2019). Association of step volume and intensity with all-cause mortality in older women. JAMA Int Med.

[52] Ekelund U, Tarp J, Steene-Johannessen J (2019). Dose–response associations between accelerometry measured physical activity and sedentary time and all cause mortality: systematic review and harmonised meta-analysis. BMJ.

[53] Public Health England (2020). Health matters: physical activity — prevention and management of long-term conditions.

[54] Cassidy S, Fuller H, Chau J (2018). Accelerometer-derived physical activity in those with cardio-metabolic disease compared to healthy adults: a UK Biobank study of 52,556 participants. Acta Diabetol.

[55] Gobeil-Lavoie A-P, Chouinard M-C, Danish A, Hudon C (2019). Characteristics of self-management among patients with complex health needs: a thematic analysis review. BMJ Open.

[56] Nolan CM, Longworth L, Lord J (2016). The EQ-5D-5L health status questionnaire in COPD: validity, responsiveness and minimum important difference. Thorax.

[57] Zanini A, Aiello M, Adamo D (2015). Estimation of minimal clinically important difference in EQ-5D visual analog scale score after pulmonary rehabilitation in subjects with COPD. Respir Care.

[58] Pickard AS, Neary MP, Cella D (2007). Estimation of minimally important differences in EQ-5D utility and VAS scores in cancer. Health Qual Life Outcomes.

[59] Hoehle LP, Phillips KM, Speth MM (2019). Responsiveness and minimal clinically important difference for the EQ-5D in chronic rhinosinusitis. Rhinology.

[60] Craig P, Dieppe P, Macintyre S (2013). Developing and evaluating complex interventions: the new Medical Research Council guidance. Int J Nurs Stud.

[61] Pawson R, Tilley N (1997). Realistic evaluation.

[62] Harris SM, Joyce H, Miller A (2018). The attitude of healthcare professionals plays an important role in the uptake of diabetes self-management education: analysis of the Barriers to Uptake of Type 1 Diabetes Education (BUD1E) study survey. Diabet Med.

[63] Paterick TE, Patel N, Tajik AJ, Chandrasekaran K (2017). Improving health outcomes through patient education and partnerships with patients. Proc (Bayl Univ Med Cent).

